# Behandlung dissoziativer Symptome mit Nalmefen bei Patienten mit Borderline-Persönlichkeitsstörung und komplexer posttraumatischer Belastungsstörung

**DOI:** 10.1007/s00115-021-01239-1

**Published:** 2021-12-03

**Authors:** Frank Enning, Christian Schmahl

**Affiliations:** Klinik für Psychosomatik und Psychotherapeutische Medizin, Zentralinstitut für Seelische Gesundheit, Medizinische Fakultät Mannheim, Universität Heidelberg, J5, 68159 Mannheim, Deutschland

## Hintergrund

Dissoziative Phänomene einschließlich Flashbacks und dissoziative Anfälle sind häufige und therapiehemmende Probleme bei Patienten mit Borderline-Persönlichkeitsstörung und komplexer Posttraumatischer Belastungsstörung (PTBS). Es wird angenommen, dass das Endogene Opioidsystem (EOS) dabei eine wichtige Rolle spielt. Bezüglich des EOS wurde bisher nur Naltrexon und sein Wirkmechanismus bei der Behandlung dissoziativer Symptome untersucht. Nalmefen ist ein weiterer Opioid-Antagonist und wir untersuchten seine Wirkung im Rahmen eines individuellen Behandlungsversuchs bei Patienten mit Borderline-Persönlichkeitsstörung, komplexer PTBS und schweren dissoziativen Symptomen. Nalmefen zeigte vielversprechende Ergebnisse als mögliche Alternative zu Naltrexon, insbesondere bei Unverträglichkeit oder unzureichender Wirksamkeit.

Ein klinisch relevantes Symptomcluster der Borderline-Persönlichkeitsstörung (BPS) und der komplexen PTBS ist die Dissoziation, die im DSM-Item „Vorübergehende, stressbedingte paranoide Vorstellungen oder schwere dissoziative Symptome“ enthalten ist. Zu den dissoziativen Phänomenen gehören Derealisation, Depersonalisation, Analgesie und Veränderungen der Wahrnehmung [1]. Nichtsuizidale Selbstverletzungen werden häufig eingesetzt, um diese Phänomene zu beenden. Das EOS ist an der Vermittlung von Stressreaktionen und an endogenen analgetischen Mechanismen beteiligt [2]. Die Exposition gegenüber Stress kann zur Freisetzung von Opioiden und zur Entwicklung einer stressinduzierten Analgesie führen [3]. Naltrexon kann diese Analgesie bei Patienten mit posttraumatischer Belastungsstörung (PTBS) blockieren [4]. Es gibt mehrere Fallberichte über den erfolgreichen Einsatz von Naltrexon zur Behandlung von Selbstverletzungen und Flashbacks [5–7]. In einer offenen Studie wurde ein signifikanter Rückgang der dissoziativen Symptome bei Patienten mit BPD unter Behandlung mit Naltrexon beobachtet [8]. Eine kleine randomisiert-kontrollierte Studie konnte jedoch keine signifikante Wirkung von Naltrexon bei dissoziativen Symptomen bestätigen [9]. Nalmefen zeigt sowohl gemischte agonistische als auch antagonistische Effekte am κ‑Opioid-Rezeptor. Die antagonistische Wirkung besteht in seiner Fähigkeit, die dysphorisierende Wirkung des κ‑Dynorphin-Liganden zu blockieren [10]. Eine Wirkung höherer Dosen von Nalmefen zur Behandlung dissoziativer Symptome bei der PTBS wurde bereits früher berichtet [11].

## Falldarstellung

Eine 22-jährige Studentin mit diagnostizierter BPS (ICD-10: F60.31) und PTBS (ICD: F43.1), nach körperlicher und sexueller Gewalt durch den Vater vom 4. bis zum 18. Lebensjahr, litt neben starken Stimmungsschwankungen, Leeregefühlen, Suizidideationen, Selbstverletzungen durch Verbrühen, Intrusionen und Alpträumen an massiven dissoziativen Symptomen. Neben einer Medikation mit L‑Thyroxin 75 µg/Tag bei latenter Hypothyreose wurde der Patientin aufgrund täglicher dissoziativer Anfälle Valproinsäure in einer Dosierung von 1000 mg/Tag verabreicht, was keine signifikanten Effekte zeigte. Zu Beginn der ambulanten psychiatrischen Behandlung berichtete die Patientin über tägliche dissoziative Anfälle. Diese Symptome machten es ihr unmöglich, eine Tagesstruktur aufzubauen oder ihre Berufsausbildung zu absolvieren. Auch andere Medikationsversuche mit Sertralin und Tiaprid waren erfolglos gewesen.

**Figure 1:**
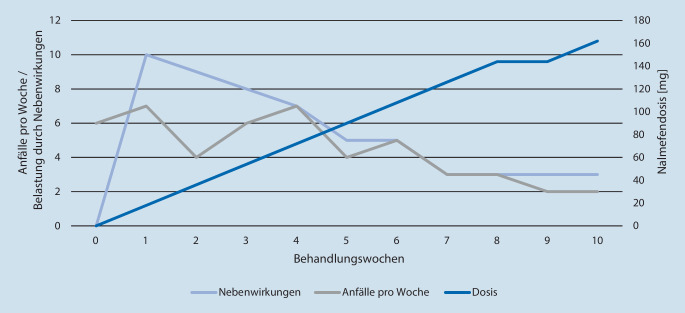

Nalmefen wurde mit 18 mg/Tag begonnen und im Laufe der Behandlung auf insgesamt 162 mg/Tag gesteigert. Wie bereits früher beschrieben [11], zeigte die Patientin bei niedriger Dosierung erhebliche Nebenwirkungen (z. B. Kopfschmerzen, Übelkeit und Schwindel), die mit Erhöhung der Dosis nachließen und bei 162 mg/Tag fast vollständig aufhörten. Gleichzeitig kam es zu einer deutlichen Reduktion der dissoziativen Anfälle, sowohl in der Häufigkeit als auch in der Dauer und Intensität (Abb. 1).

Neben dieser Patientin verabreichten wir Nalmefen bei 16 stationären Patientinnen mit BPS und komplexer PTBS und ausgeprägter dissoziativer Symptomatik. Bei 4 Patientinnen musste die Behandlung aufgrund der oben beschriebenen Nebenwirkungen bei einer Dosis von 18 mg/Tag, bei einer weiteren Patientin bei einer Dosis von 36 mg/Tag abgebrochen werden. Bei 4 Patientinnen konnte die Behandlung mit 36 mg/Tag ohne nennenswerte Nebenwirkungen durchgeführt werden. In 5 Fällen wurde die Behandlung mit 54 mg ohne nennenswerte Nebenwirkungen durchgeführt. In 2 Fällen traten bei 18 mg/Tag deutliche Nebenwirkungen in Form von Übelkeit und Schwindel auf, waren aber nach Erhöhung der Dosis auf 54 mg/Tag vollständig rückläufig. Von den insgesamt 17 mit Nalmefen behandelten Patientinnen zeigte sich bei 13 Patienten im Laufe der Therapie eine relevante Reduktion dissoziativer Phänomene (Tab. 1).

**Table 1:** 

Dosis	Wirkung auf dissoziative Symptomatik	Nebenwirkungen
18 mg/Tag	Dtl. Besserung	Keine
54 mg/Tag	Dtl. Besserung	Unter 18 mg UAW, unter 54 mg/Tag keine UAW
54 mg/Tag	Dtl. Besserung	Unter 18 mg UAW, unter 54 mg/Tag keine UAW
36 mg/Tag	Dtl. Besserung	Keine
54 mg/Tag	Dtl. Besserung	Keine
54 mg/Tag	Kaum Besserung	Keine
36 mg/Tag	Dtl. Besserung	Keine
72 mg/Tag	Dtl. Besserung	Keine
36 mg/Tag	Dtl. Besserung	Keine
18 mg/Tag	Dtl. Besserung	Keine
36 mg/Tag	Dtl. Besserung	Keine
54 mg/Tag	Dtl. Besserung	Keine
36 mg/Tag	Abgesetzt	Übelkeit u. Schwindel
18 mg/Tag	Abgesetzt	Übelkeit u. Schwindel
18 mg/Tag	Abgesetzt	Übelkeit u. Schwindel
18 mg/Tag	Abgesetzt	Übelkeit u. Schwindel

## Diskussion und Schlussfolgerungen

Die Wirkung des Opioidrezeptorantagonisten Nalmefen bei Patientinnen mit BPS und ausgeprägten dissoziativen Symptomen unterstützt die Hypothese einer Beteiligung des endogenen Opioidsystems an diesen Phänomenen [12]. Unsere Ergebnisse stehen im Einklang mit denen, die bereits früher bei Veteranen mit PTBS berichtet wurden [11]. Im Gegensatz zu Naltrexon scheint Nalmefen eine paradoxe Dosis-Nebenwirkungs-Beziehung in relevantem Ausmaß zu verursachen. Dies ist eine Implikation, die bei der klinischen Anwendung dieser Substanz beachtet werden sollte. Da dem Symptomkomplex Dissoziation eine zentrale Rolle als Prädiktor für den Erfolg der Psychotherapie der BPS und der komplexen PTBS zukommt [13, 14], stellen Opioidrezeptorantagonisten in einem multimodalen therapeutischen Ansatz eine wichtige medikamentöse Therapieoption dar [15]. Hier kann Nalmefen als Alternative zu Naltrexon gesehen werden, insbesondere bei Unverträglichkeit oder unzureichender Wirksamkeit.

## References

[1] Krystal JH, Bennett AL, Bremner JD, Southwick SM, Charney DS, Friedman MJ, Charney DS, Deutsch AY (1995). Toward a cognitive neuroscience of dissociation and altered memory functions in posttraumatic stress disorder. Neurobiological and clinical consequences of stress.

[2] Amir S, Brown ZW, Amit A (1980). The role of endorphins in stress: evidence and speculations. Neurosci Biobehav Rev.

[3] Terman GW, Shavit Y, Lewis JW, Cannon JT, Liebeskind JC (1984). Intrinsic mechanisms of pain inhibition: activation by stress. Science.

[4] Roth AS, Ostroff RB, Hoffman RE (1996). Naltrexone as a treatment for repetitive self-injurious behavior: an open-label trial. J Clin Psychiatry.

[5] Winchel RM, Stanley M (1991). Self-injurious behavior: a review of the behavior and biology of self-mutilation. Am J Psychiatry.

[6] Pitman RK, van der Kolk BA, Orr SP, Greenberg MS (1990). Naloxone-reversible analgesic reponse to combat-related stimuli in post traumatic stress disorder. Arch Gen Psychiatry.

[7] Bills LJ, Kreisler K (1993). Treatment of flashbacks with naltrexone. Am J Psychiatry.

[8] Schmahl C, Stiglmayr C, Böhme R, Bohus M (1999). Behandlung von dissoziativen Symptomen bei Borderline-Persönlichkeitsstörungen mit Naltrexon. Nervenarzt.

[9] Schmahl C, Kleindienst N, Limberger M, Ludäscher P, Mauchnik J, Deibler P, Brünen S, Hiemke C, Lieb K, Herpertz S, Reicherzer M, Berger M, Bohus M (2012). Evaluation of naltrexone for dissociative symptoms in borderline personality disorder. Int Clin Psychopharmacol.

[10] Bart G, Schluger JH, Borg L, Ho A, Bidlack JM, Kreek MJ (2005). Nalmefene induced elevation in serum prolactin in normal human volunteers: partial kappa opioid agonist activity?. Neuropsychopharmacology.

[11] Glover H (1993). A preliminary trial of Nalmefene for the treatment of emotional numbing in combat veterans with post-traumatic stress disorder. Isr. J. Psychiatry Relat. Sci..

[12] Bandelow B, Schmahl C, Falkai P, Wedekind D (2010). Borderline personality disorder: a dysregulation of the endogenous opioid system?. Psychol Rev.

[13] Kleindienst N, Limberger MF, Ebner-Priemer UW, Keibel-Mauchnik J, Dyer A, Berger M, Schmahl C, Bohus M (2011). Dissociation predicts poor response to dialectial behavioral therapy in female patients with borderline personality disorder. J Pers Disord.

[14] Kleindienst N, Priebe K, Görg N, Dyer A, Steil R, Lyssenko L, Winter D, Schmahl C, Bohus M (2016). State dissociation moderates response to dialectical behavior therapy for posttraumatic stress disorder in women with and without borderline personality disorder. Eur J Psychotraumatol.

[15] Timaus C, Meiser M, Wiltfang J, Bandelow B, Wedekind D (2021). Efficacy of naltrexone in borderline personality disorder, a retrospective analysis in inpatients. Hum Psychopharmacol.

